# COVID‐19 in a case with Kikuchi‐Fujimoto disease

**DOI:** 10.1002/ccr3.3748

**Published:** 2021-01-05

**Authors:** Kaveh Jaseb, Najmeh Nameh Goshay Fard, Nima Rezaei, Siavash Sadeghian, Saeid Sadeghian

**Affiliations:** ^1^ Thalassemia & Hemoglobinopathy Research Center Health Research Institute Ahvaz Jundishapur University of Medical Sciences Ahvaz Iran; ^2^ Research Center for Immunodeficiencies, Children’s Medical Center Tehran University of Medical Sciences Tehran Iran; ^3^ Department of Immunology, School of Medicine Tehran University of Medical Sciences Tehran Iran; ^4^ Network of Immunity in Infection, Malignancy and Autoimmunity (NIIMA) Universal Scientific Education and Research Network (USERN) Tehran Iran; ^5^ Ahvaz Jundishapur University of Medical Sciences Ahvaz Iran; ^6^ Department of Paediatric Neurology, Golestan Medical, Educational, and Research Centre Ahvaz Jundishapur University of Medical Sciences Ahvaz Iran

**Keywords:** cervical lymphadenopathy, fever, Kikuchi‐Fujimoto disease, novel coronavirus disease 2019, skin rash

## Abstract

The accurate diagnosis of Kikuchi‐Fujimoto disease can protect children from unnecessary diagnostic procedures and treatments. Also, the co‐occurrence of rare diseases with other diseases can improve or worsen the symptoms of the patients.

## Graphical abstract

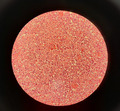

## 1 INTRODUCTION

Kikuchi‐Fujimoto disease (KFD) or histiocytic necrotizing lymphadenitis is a rare and self‐limiting benign disease.^1^ KFD is mostly seen in young adults before the age of 30 years with a female predominance,^2^ but in children, it is more common in boys.^1^ KFD is typically characterized by cervical lymphadenopathy, fever, and skin rash.^3^ The etiology of KFD is still unknown, and it can lead to misdiagnosis with other diseases, including lymphoma, systemic lupus erythematosus, or hematological disorders.^4^ Although supportive therapy is usually enough for management of KFD,^2^ the misdiagnosis can impose patients to unnecessary diagnostic procedures and treatments. In this study, we present a case of Kikuchi‐Fujimoto disease that was also positive for novel coronavirus disease 2019 (COVID‐19).

## 2 CASE REPORT

**FIGURE 1:**
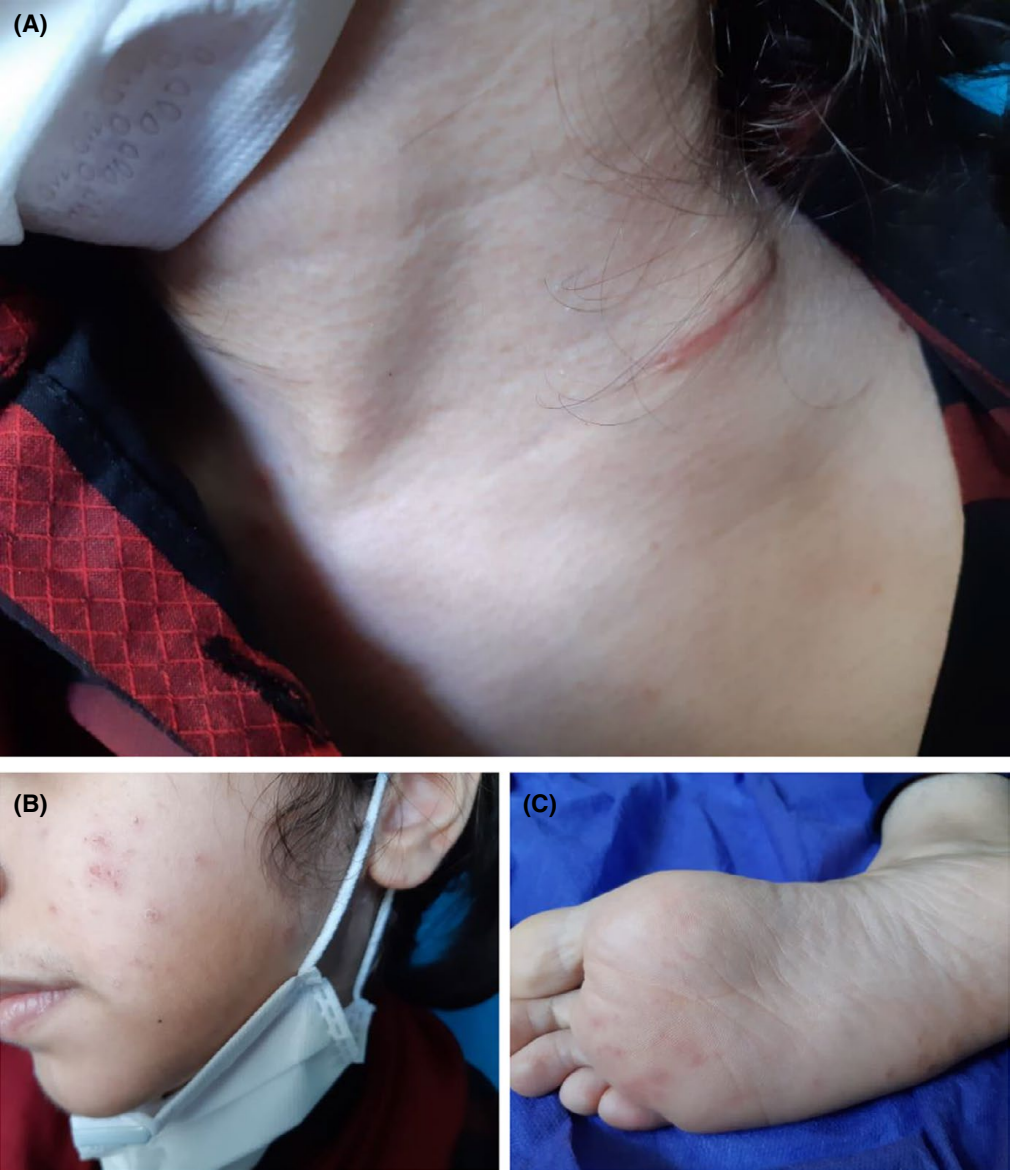
Initial clinical manifestations of patient with Kikuchi‐Fujimoto disease. A, Cervical lymphadenopathy. B, Face erythematous plaques. C, Foot erythematous plaques

A 16‐year‐old girl was admitted to the hospital with a 6‐month history of left cervical lymphadenopathy. At the beginning of the disease, the lymph nodes were painless with 0.5 cm size, but over time, they became mobile, rubbery, painful, and larger (About 2.5 cm) (Figure 1A). Three months after initial of symptoms, she had frequent fevers, night sweats, myalgia, and weight loss. One month later, the patient complained of hair loss and erythematous plaques on the face, limbs, and hands (Figure 1B‐C). The patient did not have any history of autoimmune or infectious disease before symptom onset.

Complete blood count showed elevated erythrocyte sedimentation rate (ESR, 98 mm/h), lactate dehydrogenase (LDH, 865 IU/L), and C‐reactive protein (CRP, 25 mg/dL). On the other hand, white blood count (WBC, 3600/mcL), red blood count (RBC, 3.3 million/mcL), hemoglobin (HB, 9 g/dL), and hematocrit were decreased. Immunoassay for autoimmune disease was 50 IU/mL for antidouble stranded DNA (anti‐dsDNA) and 1/1000 titer for antinuclear antibody (ANA). Serology test was negative for rheumatoid factor (RF), human immunodeficiency virus (HIV), and hepatitis B and C. Blood culture to detect bacteria, fungi, or other common germs was also negative.

**FIGURE 2:**
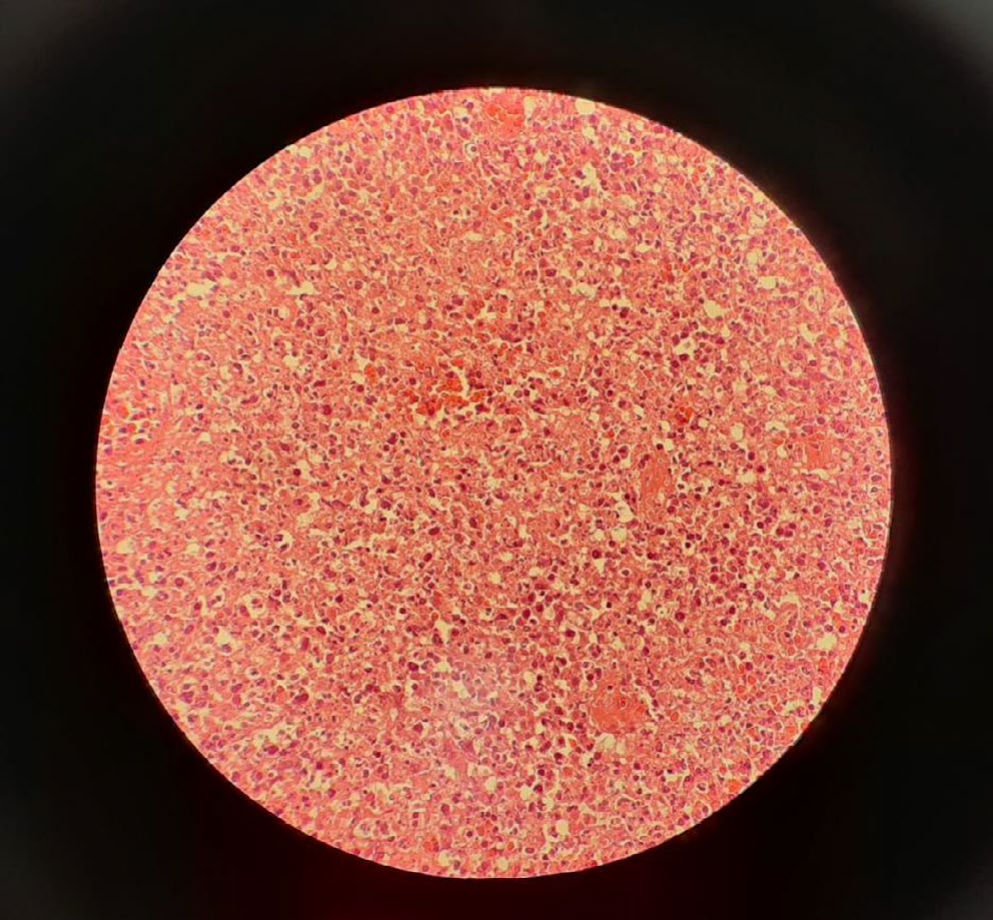
A core needle biopsy was performed from a cervical lymph node and immunohistochemistry assessment showing the cores of lymphoid tissue with well‐defined paracortical necrotic lesions with nuclear debris

A core needle biopsy was performed from a 0.7 × 0.7 × 0.1 cm cervical lymph node under ultrasound guided. Immunohistochemistry assessment revealed cores of lymphoid tissue with well‐defined paracortical necrotic lesions with nuclear debris. In addition, there was no infiltration of polymorphonuclear (PMN) leukocytes or plasma cells in microscopic examination (Figure 2). The diagnosis of Kikuchi‐Fujimoto disease (KFD) was made for the patient.

**FIGURE 3:**
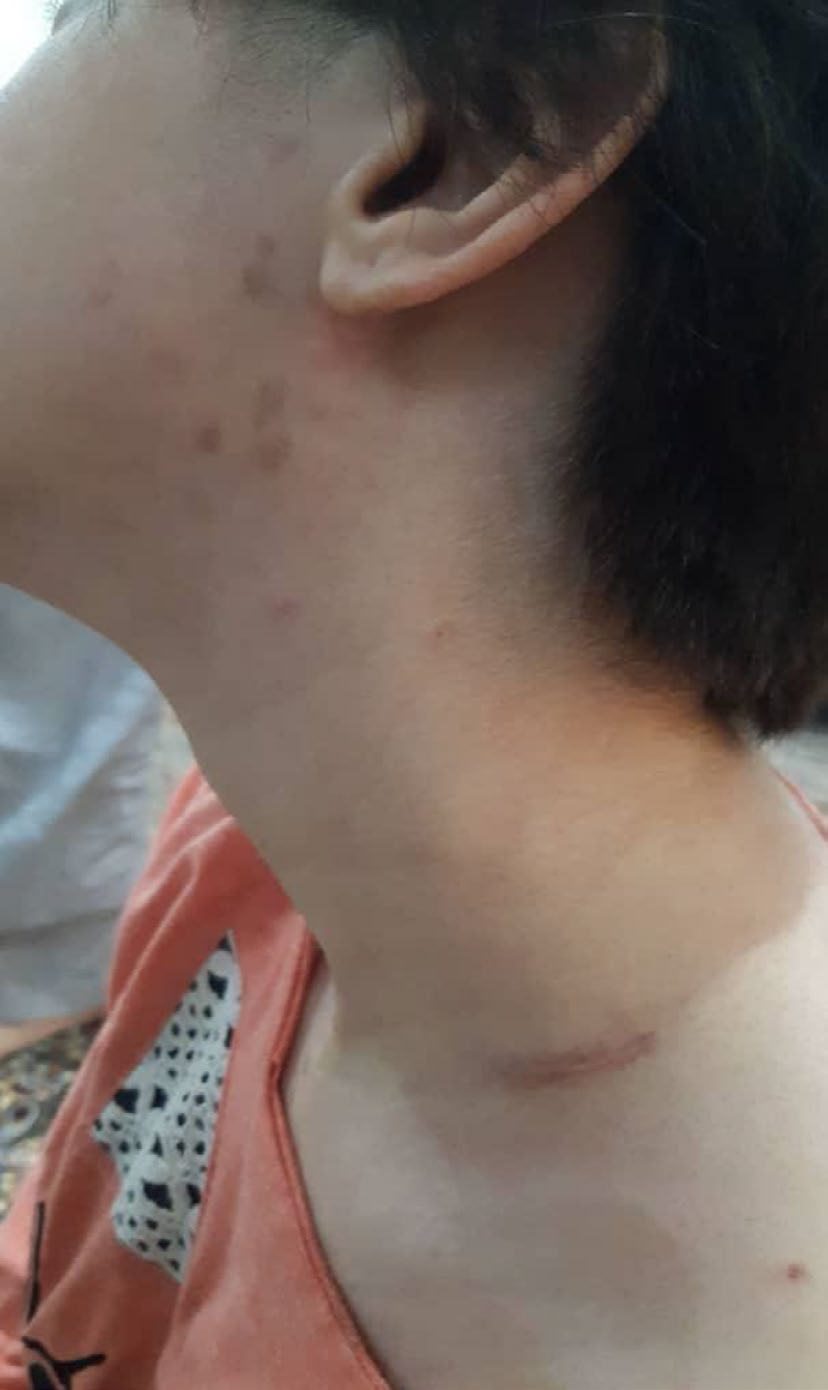
The initial clinical manifestations of patient include cervical lymphadenopathy and face erythematous plaques improved after getting infected with SARS‐CoV‐2

The patient was initially supposed to be given prednisolone, but due to fever and cough, prednisolone was refused. The patient was re‐admitted, and after PCR test, she was diagnosed with severe acute respiratory syndrome coronavirus 2 (SARS‐CoV‐2). The patient underwent supportive treatment, and interestingly after 7 days, the cervical lymphadenopathy and skin rashes significantly improved (Figure 3).

## 3 DISCUSSION

Kikuchi‐Fujimoto disease (KFD) is a histiocytic necrotizing lymphadenitis, which was described in 1972 by Kikuchi and Fujimoto in Japan.^2^ KFD is a rare and benign disease with unknown etiology.^4^ Although the etiology of disease is still unclear, viral infections (Epstein‐Barr virus, Cytomegalovirus, rhinovirus, rubella virus, and HIV)^5^ and autoimmune diseases (SLE, Polymyositis, Rheumatoid arthritis, Still’s disease, and Sjogren’s syndrome)^6^ are proposed as possible triggers for KFD.

The most common clinical presentations of KFD are lymphadenopathy (79‐94%), fever (35‐67%), cutaneous rashes (4‐32.9%), arthralgia (7‐34.1%), and hepatosplenomegaly (3‐14.8%).^2^ Less common presentations are arthritis, weight loss, loss of appetite, hepatosplenomegaly, and sweating.^4^ Our patient experienced lymphadenopathy, fevers, night sweats, myalgia, weight loss, and hair loss. The most frequent laboratory findings of KFD are elevated levels of ESR (78.9%), CRP (38.3%), and LDH (52.5%‐81.5%).^2^ Moreover, the literature has reported lymphopenia (63.8%), thrombocytopenia (5.4%‐19%), and leukocytosis (2%‐5%) in patients.^2^, ^5^ Our patient had leukopenia, severe anemia, elevated LDH, raised ESR, and CRP. The most common clinical manifestations and remarkable laboratory changes along with an excisional biopsy of involved lymph node could reveal the KFD diagnosis.

The diagnosis of KFD is based on histopathologic examination of an involved lymph node biopsy.^1^, ^4^ The most common histologic finding is the presence of areas of necrosis and apoptosis which surrounded by CD68^+^ histiocytes, CD123^+^ plasmacytoid dendritic cells, and activated CD8^+^ T‐lymphocytes.^7^ Also, the absence of neutrophils and eosinophils is an important characteristic in support of the diagnosis.^5^, ^8^ Several studies purposed that KFD may be a clinical presentation of lupus lymphadenitis or associated with infectious mononucleosis‐like syndromes such as EBV infection.^2^, ^5^, ^6^ Therefore, a complete work‐up, including precise clinical examination with an excisional biopsy, is recommended to rule out other serious autoimmune and infectious disease.

The long‐term prognosis of KFD is good, and deaths have been seen in a few patients with systemic forms of the disease.^6^, ^8^ KFD usually resolves within 1‐6 months with a 12.2% recurrence rate in children.^1^, ^6^ No specific treatment is known for KFD, and the most common approach is supportive therapy.^2^, ^4^ NSAIDs are used to relieve some of the localized signs and symptoms such as fever and tenderness of lymph nodes. In severs forms of the disease, patients maybe benefit from corticosteroid therapy, hydroxychloroquine, or intravenous immunoglobulin.^4^, ^8^

To our best knowledge, this is the first reported case of KFD, who was affected by COVID‐19. Although the association of KFD and COVID‐19 cannot be confirmed in this case, considering some cutaneous manifestations of COVID‐19, such condition should not be missed.

## CONFLICT OF INTEREST

The authors have no conflicts of interest in this study.

## 4 INFORMED CONSENT

A written consent has been obtained from the patient.

## AUTHOR CONTRIBUTIONS

Dr. Kaveh has been responsible for identifying the disease in the patient and collecting patient data. Dr. Nameh Goshay Fard has been responsible for collecting patient data. Dr. Rezaei has been responsible for modified the content of the work and the structure of the manuscript. Dr. Siavash Sadeghian participated in writing manuscript and collecting photos. Dr. Saeid Sadeghian has participated in writing of the manuscript and has been the coordinator of this research. He is also responsible for submitting and answering journal questions.

## ETHICAL APPROVAL

The study was under the ethical standards of the local ethics committee.

## Data Availability

The data that support the findings of this study are available on request from the corresponding author.

## References

[1] Selvanathan SN , Suhumaran S , Sahu VK , Chong CY , Tan NW , Thoon KC . Kikuchi‐Fujimoto disease in children. J Paediatr Child Health. 2020;56(3):389–393. 31576642 10.1111/jpc.14628

[2] Lin YC , Huang HH , Nong BR , et al. Pediatric Kikuchi‐Fujimoto disease: A clinicopathologic study and the therapeutic effects of hydroxychloroquine. J Microbiol Immunol Infect. 2019;52(3):395–401. 29050748 10.1016/j.jmii.2017.08.023

[3] Shabana M , Warnack W . An atypical neurologic complication of Kikuchi‐Fujimoto Disease. Neurol Neuroimmunol Neuroinflamm. 2020;7(3):e707. 32179622 10.1212/NXI.0000000000000707 PMC7136040

[4] Lelii M , Senatore L , Amodeo I , et al. Kikuchi‐Fujimoto disease in children: two case reports and a review of the literature. Ital J Pediatr. 2018;44(1):83. 30021595 10.1186/s13052-018-0522-9 PMC6052688

[5] Singh JM , Shermetaro CB . Kikuchi‐Fujimoto disease in Michigan: a rare case report and review of the literature. Clin Med Insights Ear Nose Throat. 2019;12:1179550619828680. 30833818 10.1177/1179550619828680 PMC6393831

[6] Baziboroun M , Bayani M , Kamrani G , Saeedi S , Sharbatdaran M . Kikuchi‐Fujimoto disease in an Iranian woman; a rare but important cause of lymphadenopathy. Arch Acad Emerg Med. 2019;7(1):e3. 30847438 PMC6377216

[7] Joean O , Thiele T , Raap M , Schmidt RE , Stoll M . Take a second look: it’s Kikuchi’s disease! A case report and review of literature. Clin Pract. 2018;8(4):1095. 30631410 10.4081/cp.2018.1095 PMC6297864

[8] Lame CA , Loum B , Fall AK , Cucherousset J , Ndiaye AR . Kikuchi‐Fujimoto disease, a rare cause of lymphadenopathy in Africa. Description of the first case in Senegal and review of the literature. Eur Ann Otorhinolaryngol Head Neck Dis. 2017;134(5):347–349. 28279633 10.1016/j.anorl.2017.02.007

